# Establishment of an *In Vitro* System Representing the Chicken Gut-Associated Lymphoid Tissue

**DOI:** 10.1371/journal.pone.0049188

**Published:** 2012-11-19

**Authors:** Noorjahan Banu Alitheen, Susan Jane McClure, Swee Keong Yeap, Ye Wen Kristeen-Teo, Sheau Wei Tan, Peter McCullagh

**Affiliations:** 1 Department of Cell and Molecular Biology, Faculty of Biotechnology and Biomolecular Sciences, Universiti Putra Malaysia, Serdang, Selangor, Malaysia; 2 F.D. McMaster Laboratory, CSIRO Livestock Industries, Armidale, Australia; 3 Institute of Bioscience, Universiti Putra Malaysia, Serdang, Selangor, Malaysia; 4 John Curtin School of Medical Research, Australian National University, Canberra, Australia; University of Pittsburgh, United States of America

## Abstract

The bursa of Fabricius is critical for B cell development and differentiation in chick embryos. This study describes the production *in vitro*, from dissociated cell suspensions, of cellular agglomerates with functional similarities to the chicken bursa. Co-cultivation of epithelial and lymphoid cells obtained from embryos at the appropriate developmental stage regularly led to agglomerate formation within 48 hours. These agglomerates resembled bursal tissue in having lymphoid clusters overlaid by well organized epithelium. Whereas lymphocytes within agglomerates were predominantly Bu-1a^+^, a majority of those emigrating onto the supporting membrane were Bu-1a^−^ and IgM^+^. Both agglomerates and emigrant cells expressed activation-induced deaminase with levels increasing after 24 hours. Emigrating cells were actively proliferating at a rate in excess of both the starting cell population and the population of cells remaining in agglomerates. The potential usefulness of this system for investigating the response of bursal tissue to avian Newcastle disease virus (strain AF2240) was examined.

## Introduction

Avian B cell development occurs in the bursa of Fabricius, a specialized organ with a follicular structure of mesenchymal and epithelial elements [1]. The primary role of the bursa of Fabricius, officially called the bursa cloacalis, is to function as the central locus of B cell development [2]. Bursal follicular development is induced by immigration of lymphoid stem cells, some of splenic origin, through mesenchyme to reach the bursal epithelium where they undergo proliferative expansion [3]. The Bu-1 antigen is one of the earliest antigens to appear in B cell ontogeny and is expressed on virtually all B cells except for plasma cells [4], [5]. It thus serves as a marker for stage of maturation of B cells. Siatskas and Boyd [6] reported that the Bu-1 antigen is a marker which identifies B-cell precursors with pre-bursal stem cell activity. B cell development in the bursa of Fabricius is dependent on the presence of an epithelial reticulum [7]. Consistent with this, bursal epithelium has been reported to be necessary if immigrant stem cells are to proliferate and commence gene conversion [3].

The bursa is a target for several avian viruses including infectious bursal disease virus (IBDV), Marek's disease and Newcastle Disease virus (NDV) [8], [9]. In the case of IBDV, studies have shown that the target cells are the B lymphocytes and virus replication leads to extensive bursal follicle destruction [10].

Whereas *in vivo* systems are mandatory for studying migratory patterns within the lymphoid system, *in vitro* systems which permit continuous observation and experimental manipulation can complement understanding of both normal development and host responses to pathogens. Although *in vitro* analogues of mammalian B cell development exist [11], [12] comparable avian systems are lacking even though B cell development in the chick bursa was described before extrapolation of this observation to mammals. This study aimed to develop an *in vitro* system which mimics avian bursal B cell development to permit investigation of the response to relevant pathogens.

## Materials and Methods

### Ethics Statement

All experiments were conducted in accord with the guidelines of laboratory animal care of Universiti Putra Malaysia, (Ref: UPM Research Policy). Approval of the ethics committee is not needed for work carried out in chicken embryos before the time of hatching, (Ref: UPM/FPV/PU/B901).

### Agglomerate Culture

**Figure 1:**
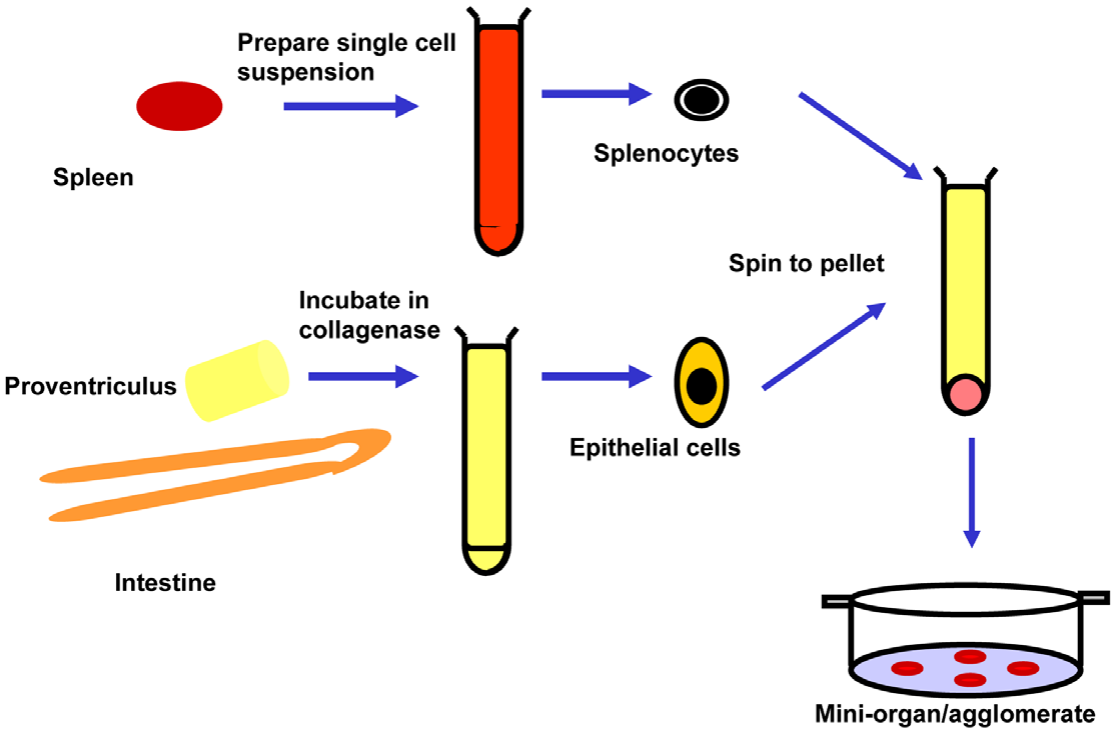
Preparation of chicken epithelium-lymphocytes agglomerates.

Embryos used were from an outbred broiler strain and all fertilized eggs were incubated at 38°C. Single cell suspensions were prepared from spleen and a mixture of proventriculus and intestine from the same individual 15–20 day chick embryos (to avoid any confounding effects of allogeneic interactions) by enzymatic disaggregation. Briefly, proventriculus and intestine were minced and incubated in 2000 Unit/ml collagenase (Sigma, USA) in PBS supplemented with 0.1% BSA and 0.6% sodium citrate (PBS-BSA-SC) for 30 minutes at 37°C. After three washes in (PBS-BSA-SC) the epithelial cells were suspended in 2 ml of PBS-BSA-SC. The spleen was disrupted, using a wire mesh, in PBS-BSA-SC. The resulting cell suspension was centrifuged at 200*×* g for 10 min and suspended in 2 ml of PBS-BSA-SC. Cells in the suspensions were counted. After removal of larger fragments, a mixture (1∶1) of epithelial cells and spleen cells was pelleted by centrifugation. Dispersed pellets were deposited as drops (∼4 mm diameter) on a 0.4 mm membrane incorporated in a cell culture insert (3090, Falcon)(BD, USA) which was placed in a 6 well dish (3502, Falcon) (BD, USA) containing pre-warmed (37°C) Dulbecco's modified Eagle's medium (DMEM) (Sigma, USA) supplemented with 5% fetal calf serum (FCS), 1% HEPES and 100 U penicillin G/ml and 100 mg streptomycin/ml (all from Sigma, USA). Cultures were incubated at 37°C in 5% CO_2_ with replacement by fresh pre-warmed media after 48 hours. Cultures were photographed live after 5 days before fixation in buffered formaldehyde. The protocol for preparation of the epithelium-lymphocyte agglomerates is summarized in Fig. 1. Agglomerates were embedded, sectioned and stained with Hematoxylin and Eosin (H&E) [13].

### Detection of proliferation of preculture, agglomerate and emigrant cells

Cells adherent to the membrane were fixed in 3% glutaraldehyde in PBS for 1 hour and stained with immunoperoxidase using the Ultravision Detection System Anti-polyvalent, HRP/DRB Ready to Use kit (Lab Vision Corporation, CA, USA) with purified mouse anti-human Ki-67 monoclonal antibody (556003, BD Bioscience). In addition to immunoperoxidase staining, the proliferation index of mixtures of freshly isolated cells, cultured agglomerates and cultured emigrant cells were compared using a BrdU Cell Proliferation Kit (Chemicon, USA). Briefly, samples of 5×10^4^ cells from each of the pre-cultivation mixture of proventriculus and intestine with splenocytes, dissociated agglomerates and emigrant cells were suspended in 200 µL of Dulbecco's modified Eagle's medium (DMEM) supplemented with 5% fetal calf serum (FCS), 1% HEPES and 100 U penicillin G/ml and 100 mg streptomycin/ml and added to a 96 well plate (BD Biosciences, USA). Twenty µL of BrdU reagent was added to all wells, except the unstained control. Cultures were incubated for a further 16 hours and then pelleted. The contents of each well were fixed and washed before BrdU detection antibody was added. Thereafter, the culture was washed and goat anti-mouse IgG, peroxidase labeled conjugate was added. The conjugate was targeted with 3,3′,5,5″-tetramethylbenzidine (TMB) peroxidase substrate in the dark for staining. Finally, 2.5 N sulfuric acid stop solution was added and the plate was read at 450 nm wavelength using a μ Quant ELISA Reader (Bio-Tek Instruments, USA). The proliferation index of emigrant cells, was compared with that of both the pre-cultivation mixture of proventriculus and intestine with splenocytes and the dissociated agglomerate. It was calculated using the following formula:
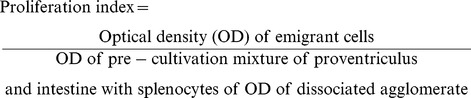



### Proliferation study of agglomerate and emigrant splenocytes using CFSE labelling

InVitrogen CellTrace CFSE kit was used to investigate the proliferation of lymphocytes in the cultured splenocytes, agglomerate and emigrant cells. Before establishment of the *in vitro* system of chicken lymphoid tissues, only lymphocytes (10^6^ cells/ml) from spleen of 15 day chick embryo were resuspended in prewarmed PBS/0.1% BSA and stained with CFSE at the concentration of 10 µM. The suspension was then incubated at 37°C for 10 minutes followed by addition of 5 volumes of ice-cold medium on ice for 5 minutes to quench the staining. Excess dye was removed by washing twice with DMEM supplemented with 5% FCS using centrifugation at 200*×* g for 10 min. Lastly the *in vitro* culture was prepared using the CFSE stained lymphocytes and unstained epithelial cells. The cells were cultured following the method and condition for agglomerate culture as previously described and harvested after 48 hours to measure stain intensity using a FACS Calibur flow cytometer (BD Biosciences, USA).

### Flow cytometry studies on fresh mixed cell population, dissociated agglomerates and emigrant cells dispersed on the membrane

A sample of 5×10^5^ cells from the pre-cultivation mixture of proventriculus and intestine with splenocytes was stained for flow cytometry as described below. Agglomerates were removed from the membrane and incubated in 500 µL PBS/BSA/EDTA containing 200 U collagenase (Sigma, USA) for 15 min to dissociate cells. All the cells were tested for viability by means of trypan blue (0.4%) staining. Emigrating cells were released from the membrane by gently scraping and suspended in PBS/BSA/EDTA. Approximately 5×10^5^ cells per sample were stained with mouse anti-chicken CD3 (Isotype control: mouse IgG_1κ_), IgM (Isotype control: mouse IgG_2bκ_) or Bu-1a-F (Isotype control: mouse IgG_1κ_) conjugated with FITC (Southern Biotech, USA). This procedure was repeated with double staining of the preculture mixture, dissociated agglomerates and emigrant cells with IgM (Isotype control: mouse IgG_2bκ_) conjugated with PE and Bu-1a-F (Isotype control: mouse IgG_1κ_) conjugated with FITC (Southern Biotech, USA). Cells were gated by forward and side scatter and by cell type specific antibodies. Gating was performed for each antibody based on appropriate isotype-stained controls. The samples were then analyzed using a FACS Calibur flow cytometer (BD Biosciences, USA). Ten thousand events per sample were acquired and cell cycle distribution was calculated using BD CellQuest™ software (BD Biosciences, USA).

### Real Time PCR detection on the expression of activation-induced deaminase (AID)

Total RNA from bursa isolated from embryos (day 18, 19, 20 and 21), agglomerate (24, 48 and 72 hours post-culture) and emigrant cells (48 and 72 hours post-culture) was extracted with TRIZOL (Invitrogen) according to the manufacturer's instructions. The purity and concentration of extracted RNA was then measured with a spectrophotometer. The detection of AID gene and 28 sRNA in embryonic bursa, agglomerate and migrating cells was carried out with two-step RT-real time PCR as described in the manufacturer's instructions. The AID gene was amplified with primers 5′- TTC CTA CGC TAC ATC TCA G-3′(forward) and 5′- CCC CTC AGG CTC AGC CTT G-3′ (reverse) [14]. Detection of 28S RNA served as a positive control in the real time PCR and the primers used were 5′- GGC GAA GCC AGA GGA AAC T-3′ (forward) and 5′- GAC GAC CGA TTT GCA CGT C-3′ (reverse) [15]. Firstly, the cDNA was synthesized using Tetro cDNA Synthesis Kit (BIOLINE). The 20 µl of reaction mixture contained a final concentration of 1× RT buffer, 0.5 mM dNTP mix, 0.5 µM of each forward and reverse primer (gene-specific primers), 10 units of MMLV reverse transcriptase, 1 unit of RNase inhibitor and 200 ng/µl of total RNA was prepared. The reaction mixture was then incubated at 45°C for 30 min followed by 85°C for 5 min. Real time PCR was performed with the SensiFast™ SYBR No-ROX Kit (BIOLINE). The reaction mixture was prepared with the final volume of 20 µl which consisted of 1× SensiFast No-ROX Mix, 0.5 µM of each forward and reverse primer (gene-specific primers) and 2 µl of cDNA. The reaction mixture was then subjected to 2 min initial incubation at 95°C, 40 cycles of 95°C for 20 s, 57°C for 20 s and 72°C for 15 s. Upon completion of the PCR amplification, the specificity of the amplified product was confirmed by melting curve analysis whereby the reaction was incubated by raising the incubation temperature from 70°C to 95°C in 0.5°C increments with a hold of 5 second at each increment.

### Viral infection of agglomerates and transmission electron microscopy

Newcastle disease virus of strain AF2240 (NDV) was propagated in allantoic fluid from 9–11 day-old embryonated chicken eggs at 37°C for 3 days. Virus was purified [16] and the titer of virus was confirmed using a haemagglutination assay [17]. Ten microliter of NDV virus (10^11^/ml embryo infection dosage) was inoculated onto each agglomerate. After 24 hours, agglomerates were fixed in 4% glutaraldehyde, washed in 0.1 M cacodylate buffer and post-fixed in 1% osmium tetroxide. Specimens were then washed in 0.1 M sodium cacodylate buffer, dehydrated through a series of acetone concentrations, infiltrated with an acetone and resin mixture and embedded in beam capsules filled with resin. Following polymerization in an oven at 60°C, ultrathin sections were cut and mounted on 200-mesh-copper grids. Sections were stained with uranyl acetate and later with lead citrate and examined using a Hitachi H-7100 transmission electron microscope (TEM).

### Statistical analysis

All data are shown as means (±SEM). Significant differences between sample means were determined using a one way ANOVA.

## Results

### Growth of 3D agglomerate

**Figure 2:**
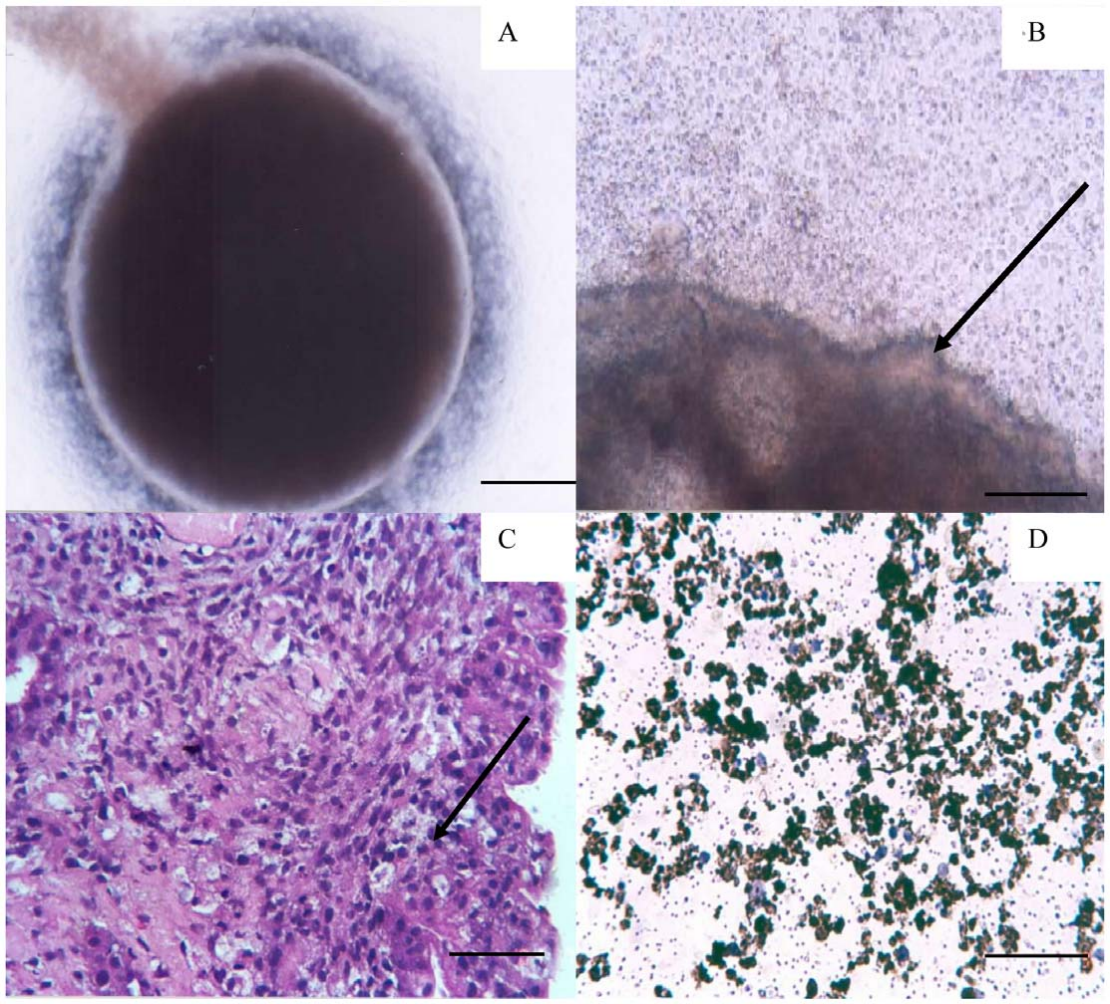
Morphological appearance of live culture and stained cells. (A) Agglomerate generated from a 15 day chick embryo – live culture photographed after 5 days. 40×. The scale bar indicates 1 mm (B) Periphery of the mini-organ. Arrow indicates the epithelial cell organization at the edge. 100×. The scale bar indicates 200 µm (C) H&E stained section of agglomerate prepared from 15 days embryonic chicken. Note columnar epithelial cells lining the marginal layer and arrow indicating cells of lymphocyte morphology in the agglomerate. 200×. The scale bar indicates 100 µm (D) Staining of cells emigrant from an agglomerate containing cells from a 15 day embryo for the presence of proliferation antigen Ki-67. Note high frequency of cells synthesizing DNA. 200×. The scale bar indicates 100 µm.

**Figure 3:**
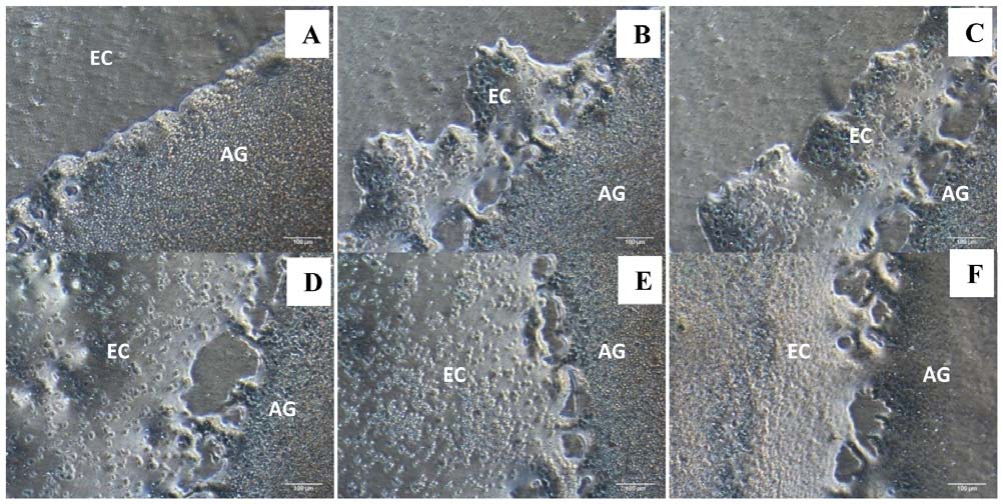
Phase contrast microscopy of migrating cells from the agglomerate. (A) day 0, (B) day 1, (C) day 2, (D) day 3, (E) day 4 and (F) day 5. 100×. AG indicates the agglomerate and EC indicates emigrate cells that migrate out from the agglomerate. Note high frequency of emigrant cells only became apparent from day 3.

Well-organized structures of approximately 3 mm diameter regularly became visible macroscopically on the culture membrane after 48 hours, provided 15–18 day embryos were used as donors (Table 1). These agglomerates contracted to approximately 2 mm diameter by 72 hours as the two cellular components coalesced (Fig. 2A) with a translucent outer layer enclosing each agglomerate (Fig. 2B). Microscopic examination of fixed H&E stained sections revealed that agglomerates were circumscribed by a well organized layer of uniformly oriented columnar epithelial cells. Associated with this were subepithelial accumulations of basophilic cells with lymphocytic morphology (Fig. 2C). Other cells with lymphocytic morphology typically formed a corona on the surrounding membrane. However, these extra-agglomerate cells were not evident at 24 hours of incubation. The spreading out/translocation of cells on the membrane was observable at 48 hours and from 72 hours onwards, the rate of migration increased markedly as evidenced by the presence of a high frequency of cells surrounding the agglomerate (Fig. 3). Hence, we recognize these cells as emigrants migrating out from the agglomerate.

**Table 1:** The age of chick embryos (days) and the number of successful replicates per number of attempts.

Embryonic age (Day)	Number of successful replicates/number of attempts
15	3/3
16	3/3
18	6/6
19	0/3
20	0/2

Each replicate used one embryonic chicken and 6 culture attempts to form agglomerates were set up in each case. The formation of at least 5 out of 6 agglomerates was required for the scoring as successful. When 19 and 20 day chicks were used, agglomerates were not formed. Each replicate used one embryonic chicken, and six mini organs were attempted for each replicate. At least 5 out of six mini organs must be formed for the attempt to count as a successful replicate. For day 19 and 20, none of the mini organs was successfully formed in all three replicates.

### Analysis of the frequency of Ki-67+ cells in the migratory population by immunoperoxidase staining

The proliferation marker, Ki-67 was used to determine whether this culture system promoted cell proliferation. A large proportion of the emigrant cell population stained brown indicating that these migratory lymphocytes were proliferating (Fig. 2D).

### Proliferation of emigrant cells

**Figure 4:**
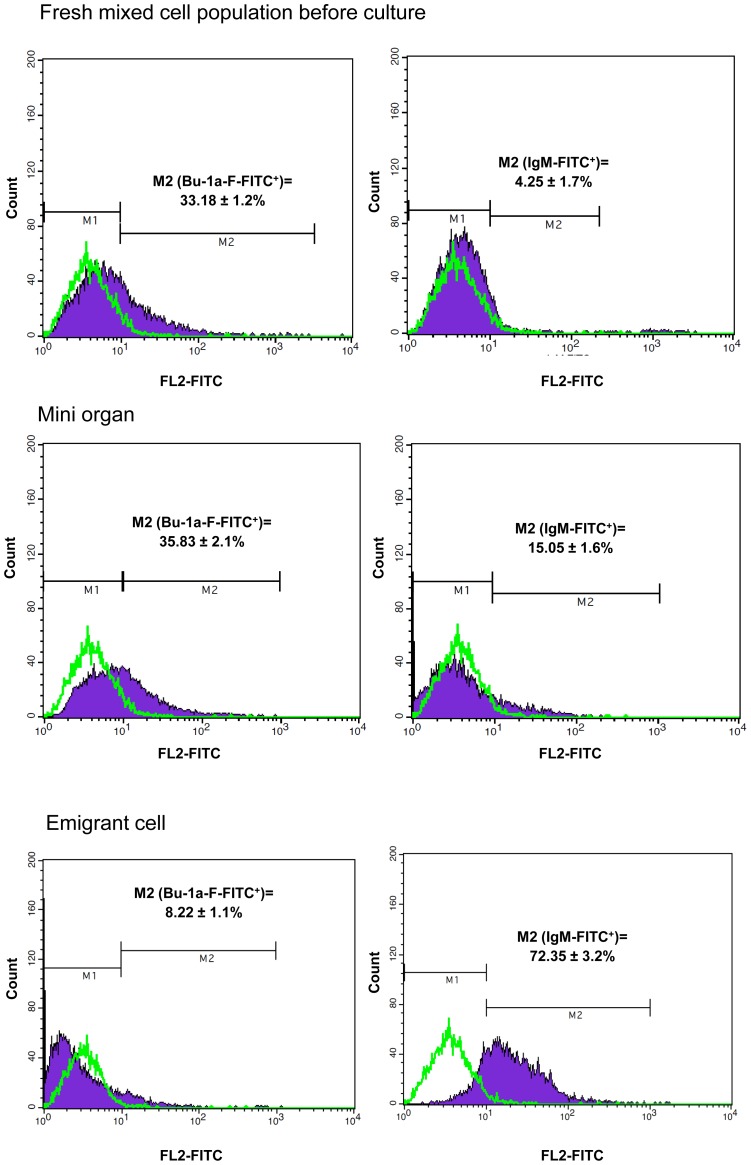
Fluorescence intensity profiles of populations from five day cultures. The percentage of fresh mixed cell population pre-culture, dissociated cultured agglomerates and cells dispersed on the membrane, expressing Bu-1a-F or IgM are shown as determined by flow cytometry. Lymphocytes were gated according to their forward and side scatter characteristics. Gating of stained lymphocytes was performed for each antibody based on appropriate isotype-stained controls. The negative result of the isotype control antibody was overlaid on the positive curve of the Bu-1a-F or IgM marker.

The proliferation indices of the cell populations are shown in Table 2. A higher rate of proliferation was detected in the emigrant cells, as indicated by the proliferation index (3.2±0.8), in comparison with both the pre-cultivation mixture and the cell suspension resulting from agglomerate disruption. In the CFSE proliferation assay, only splenocytes isolated from embryonic spleen were stained and incorporated into the agglomerate culture and splenocyte monolayer culture. After 48 hours of incubation, the agglomerate culture showed proliferation in both agglomerate and emigrant cells. The highest proliferation rate was observed in the emigrant cell population after 72 hours of incubation showing 4 cycles of division (Fig. 4). At this time point, the emigrant cells showed a higher total proliferation rate in the third and fourth generation of daughter cells (15.13±3.4%) than at the previous time point (12.54±2.8%). In the agglomerate and the splenocyte monolayer culture, the replication stopped after 2 cycles of division, thus third and fourth generation of daughter cells were not observed. This result obtained by the CFSE method is consistent with that of the BrdU assay which also showed a higher proliferation index in the emigrant cell population. The cultured splenocyte monolayer at 72 hours showed a lower proliferation at the first and second cycle of division (18.16±1.0%) when compared to that at 48 hours (29.4±2.2%).

**Table 2:** Proliferation index of emigrant cells compared to pre-cultivation mixtures of proventriculus, splenocytes and intestine or dissociated agglomerates obtained using a BrdU ELISA kit.

	Proliferation Index
Emigrant cell/pre-culture mixture	3.2±0.8
Emigrant cell/dissociated mini organ	2.7±1.3

The values were the means ± SEM of three experiments.

### B cell surface phenotype of preculture mixture, agglomerate and emigrant cells

**Figure 5:**
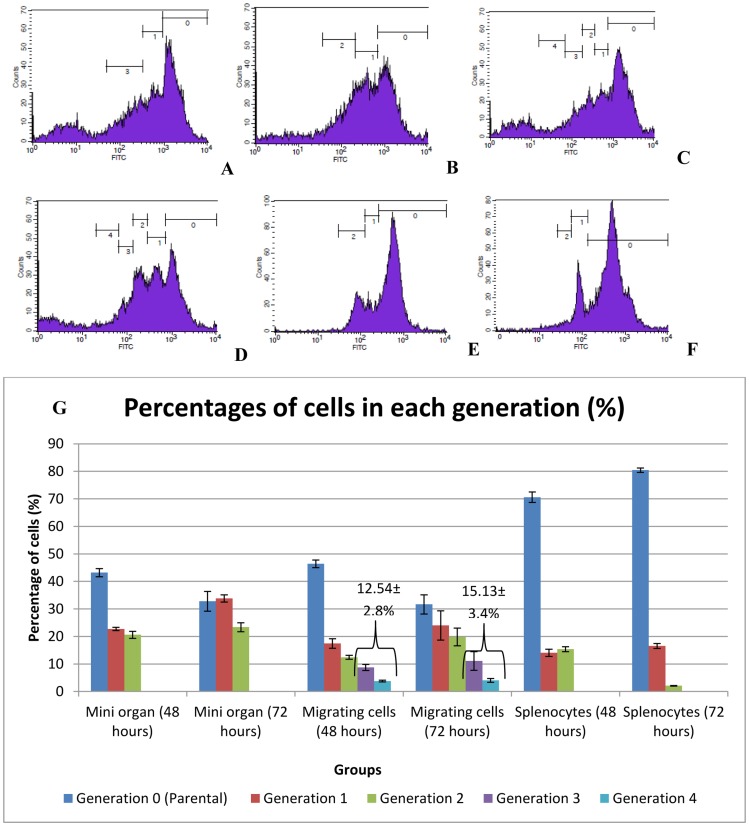
Proliferation of splenocyte analyzed by CFSE-labelling. (A) Agglomerate after 48 hours (B) Agglomerate after 72 hours (C) Emigrant cells after 48 hours (D) Emigrant cells after 72 hours (E) Splenocytes monolayer after 48 hours, (F) Splenocytes monolayer after 72 hours. Numbered peaks in the histogram indicate the number of cell divisions. One representative of three experiments is shown. (G) Summary of percentage of dividing cells of the *in vitro* system of chicken lymphoid tissue at different time points.

All of the cell populations harvested for immunophenotyping were subjected to trypan blue cell counting. The viability of the emigrant cells was 96% whilst that of agglomerate cells was 85%. The percentage of CD3^+^ T cells in agglomerates was 4% whilst that in emigrant cell populations was 6%. The flow cytometry profiles and the percentage of each population expressing IgM are shown in Fig. 5. Upon quantitative examination of IgM^+^ lymphocytes and Bu-1a-F^+^ cells, a much higher frequency of IgM^+^ cells (72.35±3.2%) was found in 5 day cultured migrating cells compared to both the preculture cells (4.25±1.7%) and agglomerates (15.05±1.6%). Double staining for IgM and Bu-1a-F indicated that a majority (61.9%) of the emigrant cells were IgM^+^ Bu-1a-F^−^. The majority of Bu-1a-F^+^ cells co-expressed IgM (9.3% as compared to 1.5% Bu-1a-F^+^ IgM^−^ cells). In contrast with the emigrant cell population, a majority of the agglomerate B cells expressed only Bu-1a-F^+^ (20.7%) and most of the IgM^+^ cells co-expressed Bu-1a (14.6%) (Table 3). In addition, double staining for IgM and Bu-1a-F on spleen cells showed that a majority of cells (69.8±2.2%) expressed Bu-1a-F^+^ IgM^−^.

**Table 3:** Double staining immunophenotyping of IgM^+^ and Bu-1a-F^+^ subpopulations in preculture mixtures, agglomerates and emigrant cells.

	Bu-1a-F^−^/IgM^−^	Bu-1a-F^−^/IgM^+^	Bu-1a-F^+^/IgM^−^	Bu-1a-F^+^/IgM^+^
**Spleen before culture**	29.4±3.1%	0.3±0.1%	69.8±2.2%	0.5±0.2%
**Fresh mixed cell population before culture**	60.3±2.7%	0.7±0.2%	38.6±1.4%	0.4±0.3%
**Mini organ**	63.5±1.8%	1.2±0.9%	20.7±1.5%	14.6±2.3%
**Emigrant cell**	27.3±3.1%	61.9±3.3%	1.5±1.1%	9.3±2.8%

The values were the mean percentages of total cell ± SEM of three experiments.

### AID expression

AID expression was monitored using real time PCR assay with 28S RNA as the house keeping gene. AID was detected in all the samples with the Cq values ranged between 25.06–32.14 (Table 4). Generally, embryonic bursa demonstrated higher expression of AID with lower Cq values as compared to both agglomerate and emigrant cells. The results also clearly indicate that the Cq values for the emigrant cells at 48 and 72 hours were lower compared to agglomerate at 24 hours.

**Table 4:** Detection of AID gene using SYBR Green I real time PCR.

Samples	Amplification of SYBR Green I real time PCR, Cq values	
	AID gene	28 sRNA
Bursa e18	25.32	17.13
Bursa e19	25.29	16.93
Bursa e20	25.22	17.14
Bursa e21	25.06	17.30
Agglomerate 24 h	32.14	16.81
Agglomerate 48 h	29.86	16.65
Agglomerate 72 h	29.72	17.19
Emigrant cells 48 h	29.67	16.71
Emigrant cells 72 h	29.65	16.63
No template control	-	Not detectable

28S RNA was used as positive control. Detection of both AID gene and 28S RNA were carried out simultaneously in real time PCR.

### NDV infection of 3D agglomerate

**Figure 6:**
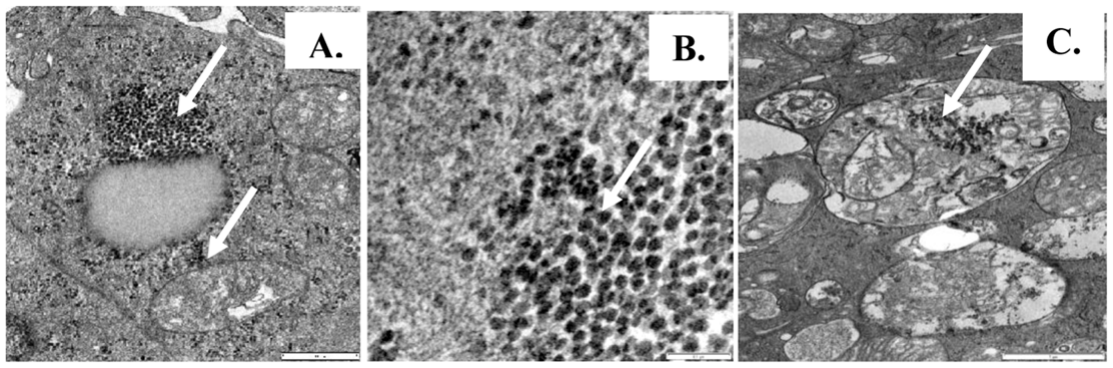
Electron micrograph of agglomerate infected with NDV virus strain AF2240. (A) Agglomerate infected for 24 hours. Arrows indicate spherical virus particles in vesicles and cytoplasm of the cells. (B) 5000× magnification of NDV particles. Note spherical particles 20–28 nm in diameter detected in the agglomerate. (C) Note virus particles in the mitochondria and damage to their structure.

Transmission electron microscopy studies undertaken 24 hours after exposure of 48 hour agglomerates to NDV revealed groups of viral particles (20–28 nm diameter) in vesicles and cytoplasm of the cells of the mini-organ (Fig. 6A and B). Some virus particles were detected in mitochondria the internal structure of which had been disrupted (Fig. 6C).

## Discussion

This report describes the development, *in vitro*, of composite bursa-like agglomerates from embryonic splenocytes and epithelial cells which sustained the proliferation and differentiation of B cells. This approach, based on the original understanding of the bursal origin, namely from an endo-mesodermal rudiment [18], employed intestine and proventriculus as a source of endodermal cells. Selection of splenic cells as a source of B cell precursors reflected their reported contribution to bursal formation *in vivo* [3]. In contrast to this original explanation of bursal origin, Nagy and Olah [19] have recently reported that only cells of ectodermal origin support bursal follicle formation within the chick embryo. Consistent with this report, we did not observe follicle formation but, nevertheless, lymphocyte differentiation occurred. We interpret this as reflecting an epithelial contribution to the *in vitro* microenvironment sufficient to facilitate some bursal functions.

Apart from supplementation of the medium with 1% HEPES, the present protocol resembled that required for development of intestinal epithelial/lymphocyte agglomerates of fetal lamb cells [18]. The occurrence of a critical cell donor age for agglomerate formation recalls a similar observation during *in vitro* modeling of fetal lamb ileal Peyer's patches [18]. The observed absence of lymphocyte/intestinal interaction *in vitro* with cells from 19 day donors is likely to reflect the reported absence of Bu-1 cells from the spleen by 19 days [21].

Two discrete populations of cells were examined, namely those remaining in the agglomerate and those emigrating from it. A high frequency of Bu-1a-F^+^ cells was found in agglomerates whereas the majority of the migrating cells were Bu-1a-F-, implying maturity, and more of these migrating cells were proliferating. Disaggregated agglomerates resembled the bursa in their majority content of *precursor* B cells [5] with even IgM^+^ B cells co-expressing Bu-1a-F, indicating immaturity. The CFSE results suggest that interaction between the embryonic splenocytes and epithelial cells sustained the proliferation of the B lymphocytes which then emigrated out from the agglomerate to its surrounding. Increased proliferation was not seen when only embryonic splenocytes were cultured in monolayer. The generation of mature proliferating B lymphocytes, evidenced by their Ki-67 expression, DNA synthesis and CFSE proliferation assay in excess of that of pre-culture or agglomerate cell populations suggested functional similarities to the bursa of Fabricius. AID is necessary for Ig gene conversion and expression of AID in the bursa is associated with diversification by gene conversion *in vivo*. Furthermore, previous studies showed that an increase in AID mRNA corresponded with the onset of extensive Ig gene conversion in the *in vivo* bursa [22]. The detection of AID in both the agglomerate and emigrant cells indicates the potential for these cells to undertake gene conversion as do those in the *in vivo* bursa. This expression increased after 24 hours, indicating that the potential for gene conversion increased after time in culture, possibly associated with increasing cell maturation. AID expression remained lower than in the *in vivo* bursa, suggesting that the efficiency of cell maturation may be lower *in vitro*.

*In vitro* cell culture can permit study of infection mechanisms under highly controlled conditions, although *in vivo* systems remain essential for studying responses to disease [23]. Given the demonstration by Petkov *et al.* [24] that a Bu-1-F^+^ and IgM^+^ subpopulation in the bursa is targeted in IBDV infections, the potential of the present agglomerate system for study of NDV infection was examined. The presence of NDV particles clustered in the agglomerate after 24 hours is consistent with productive infection and the location of viral particles within disrupted mitochondria resembles that observed following infection of chick cells with reticuloendotheliosis virus [25]. Current studies are examining the *in vitro* interaction of IBDV with agglomerates.

This report describes an *in vitro* model simulating aspects of bursal development in the embryonic chick. It is similar to one previously developed to study Peyer's patch development in fetal lambs [20]. It provides a new approach to study of the cellular interactions underlying B cell ontogeny in chickens and may afford additional opportunities to observe interactions between viruses and target cells [26], [27]. Further study should investigate whether gene conversion is taking place in the agglomerate or emigrant cell population, by attempting to demonstrate the diversification of immunoglobulin genes in the proliferating cells. Furthermore, the study of Infectious Bursa Disease Virus (IBDV) interaction with this *in vitro* agglomerate will strengthen the potential of this *in vitro* model to simulate the *in vivo* interaction.
